# Past, Present, and Future of Veterinary Epidemiology and Economics: One Health, Many Challenges, No Silver Bullets

**DOI:** 10.3389/fvets.2015.00060

**Published:** 2015-11-17

**Authors:** Andres M. Perez

**Affiliations:** ^1^College of Veterinary Medicine, University of Minnesota, St Paul, MN, USA

**Keywords:** veterinary epidemiology, one health, grand challenges, research, education, medical

Epidemiological methods are applied in veterinary science to investigate the dynamics, frequency, and determinants of diseases in populations of veterinary interest. This information is subsequently used to manipulate such determinants, with the ultimate objective of preventing, mitigating, or eliminating the impact of disease in susceptible populations (1). Because, for a given population-health problem, there are typically a number of alternative interventions available, economics, the science of making choices (2), is inherently related to veterinary epidemiology.

Epidemiologists have dedicated their work to support the prevention and control of transmissible diseases even before their infectious nature was fully understood. Cholera is one of the first documented examples in that regard; indeed, Koch postulates were not formalized until 1884–1890, following some early concepts described by Henle in 1840 (1). However, as early as in 1854, Snow conducted what in today’s nomenclature may be regarded as an “outbreak investigation.” As a result of such an investigation, he determined the relation between water obtained from a pump on Broad Street and a cholera outbreak in London. Removal of the pump contributed to the decline and eventual control of the epidemic. Although the first evidence of the pathological relation between *Vibrio cholerae* and diarrhea was recorded, coincidently, in 1854 in Italy, this early work was recognized only posthumously (3).

Approximately one decade prior to Snow’s work, in 1842, Semmelweiss established an association between certain practices and maternal mortality due to childbed fever at the General Hospital in Vienna (1). Mortality was significantly higher in rooms that were assisted by physicians who practiced necropsies the same day. Semmelweiss subsequently recommended following the same protocol implemented by those rooms with low mortality rate. That intervention, ultimately, resulted in the reduction of mortality in the exposed group to background disease levels (1). For the following 150+ years, epidemiologists have continued to use analytical methods to prevent, mitigate, and control the impact of disease even in the absence of etiological information. A recent example was the control of the bovine spongiform encephalopathy (BSE) epidemic in the U.K. in the late 1980s. Banning ruminant-derived proteins from feeding, following epidemiological evidence on association with BSE and in the absence of compelling evidence on the disease etiological agent, ultimately reduced disease incidence (4).

Snow’s and Semmelweiss’ methods were characterized by the systematic collection of data, hypothesis-driven research, use of analytical techniques, and implementation of results to inform actions and policy. Those studies and methods are considered the foundational studies of modern epidemiology. Since then, epidemiological methods have been intended to systematically investigate, and ideally quantify, the dynamics of disease in populations. I tried to integrate those consecutive, systematic steps in Figure 1.

**Figure 1:**
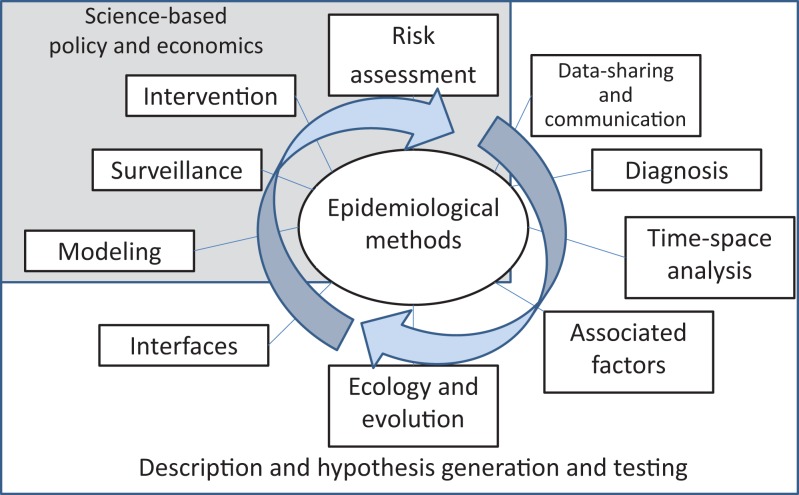
**Suggested pathway for the systematic assessment of the epidemiological dynamics of infectious diseases**.

The investigation starts with the collection of data and their communication to inform decisions. Recent developments in the field of veterinary epidemiology were related to the use of (a) *big data*, which are unusually complex databases that cannot be analyzed by traditional methods (5), and (b) hardware and software support for surveillance systems, intended to collect and analyze data and share results to inform policy in near real time (6).

As soon as the information starts to be collected, there is a need to understand the attributes and limitations of the diagnostic tests used to define health and disease status. Recent advances in the field were related to the use of latent-class models to estimate the accuracy of diagnostic tests in the field and in the absence of a gold standard (7, 8).

Disease patterns are subsequently investigated to identify temporal, spatial, and time–space relations in incidence and prevalence of disease. GIS tools, time-series analysis, and geostatistical tests have become quite popular in the field since the 1990s (9–11).

The influence of environmental, anthropogenic, and demographic factors on the levels of disease can then be quantified. A variety of toolkits and software is now available for running different types of multivariable analyses, incorporating random effects and hierarchies in relatively simple ways. Some attention has recently been put on the use of supporting vector machines for spatial and temporal analysis, and other problems relevant to veterinary epidemiology, such as multivariable analysis with a relatively large number of covariables, compared to the number of observations (12, 13).

Further assessments may look at the disease agents as dynamic organisms that evolve, change, and adapt (14). Recently, Bayesian approaches, for example, have provided a new area for development through the assessment of genetic relations and evolution accounting by the effect of time, space, and relevant epidemiological variables (15, 16).

The interfaces with other populations and systems, including the zoonotic nature of diseases, should consequently be assessed to consider potential inter-connections and relations. In recent decades, increasing attention has been put to the role that wildlife and the environment play in transmission of diseases, most notably, following the recent avian influenza pandemic, but also in relation with other pathogens (17–19).

Stages depicted in Figure 1, which, arguably, include most of the body of literature in the field of veterinary epidemiology, are essentially descriptive, intended to generate specific hypotheses, and/or aimed at testing those specific hypotheses.

The next step is the integration of information into epidemiological models of within- and between-population disease dynamics. These models may be used to assess and evaluate surveillance strategies, interventions, and assess risk of introduction into naïve populations, with the ultimate objective of informing policy (20, 21). These final stages of the analytical process (modeling, surveillance, intervention, and risk assessment) should, ideally, contribute to inform science-based policy and, for that reason, economics play a fundamental role in evaluating those alternative decisions (22, 23).

Finally, this process helps to improve our understanding of the disease and its control and to generate new hypotheses, so the descriptive and analytical process starts again through an improved process for data collection, intended to test those new hypotheses and plan for novel interventions.

There is general consensus in the community of epidemiologists in that there are more opportunities for research and work than the epidemiologists required to perform those tasks. Opportunities are vibrant, for example, in the areas of food animal health and food safety – a somewhat arbitrary distinction between pre- and post-harvest health conditions, respectively. Feeding the planet responsibly is one of the most important challenges facing humankind today. Food animals play a major role in food security as well as poverty alleviation and the control of human diseases shared with animals, referred to as zoonoses. Many of the world’s poor keep livestock are food insecure and suffer from treatable zoonotic diseases. Poor productivity, food production and distribution disequilibrium, and the neglected zoonotic diseases already are global concerns that will be aggravated by the inescapable fact of a growing world population. Indeed, based on official and private estimates from around the globe, it is projected that by the year 2050 the world population will reach the 9 billion mark, with a consequent increase in food demand and peri-urban populations (24–26).

The projection may be confounded by poverty alleviation progress, as one may argue that the growing middle class will shift food consumption patterns to a greater intake of animal proteins sourced both locally and globally. Thus, animal production is expected to progressively grow in order to fulfill the global demand (27). This situation has led to an increased recognition of the connectivity of production systems and trade at a global scale. New challenges are subsequently being faced, most notably, the increasing risk for disease epidemics and emergent diseases associated with the increase in global trade.

The One Health concept, originally attributed to Cal Schwabe, recognizes that the health of human beings, animals, and the environment is inter-connected (28). Under the One Health approach, researchers should be aware of correlations between species, groups, or ecosystems and be prepared to work together whenever relevant. A vast amount of scientific literature is available on the dynamics and impact of pathogens that are transmitted between human beings and animals. More than 200 zoonotic diseases have been described (29), including some that selectively affect marginal groups, such as bovine tuberculosis, or those that have reached pandemic proportions, with a large number of people and animals at risk worldwide, such as influenza.

I will argue that the next challenge under the One Health paradigm is the extensive use, application, and impact of applied quantitative epidemiology and economics. Economics certainly plays a role on One Health in relation to animal diseases that prevent development and sustain poverty and impact human health. For example, although sub-Saharan Africa’s share of global exports is minimum (3.5%), it was worth ~$455 billion in 2013, which is ~10 times the amount of aid the region received the same year (30). Even a minimum increase in the region participation of the share of global exports, associated with appropriate policy for sharing and distribution, would have a tremendous impact on its development. One may subsequently argue that there is a need to progressively supplement or replace aid-based approaches for development models, built upon private and public partnerships. In parallel, the volume and complexity of agricultural and livestock data available to support disease prevention and control, and production has grown to levels never seen in history over the past decade. This rapid increase in the quantity of data availability has not necessarily resulted on a consequent ability to improve the quality of our information to inform policy. I consequently argue that an emerging grand challenge is the ability to apply quantitative epidemiology and economics tools through an interdisciplinary team of agricultural, medical, and social scientists in order to improve the quality of our policy, with the ultimate objective of improving access to food and development as a mean to improve health and wealth of local and global communities.

The Epidemiology and Economics Section of Frontiers in Veterinary Science is looking forward to continue with the tradition started by Snow, Semmelweiss, and foundational figures of modern epidemiology, such as Calvin Schwabe, in contributing to preserving and promoting health in species of veterinary interest and its interface with public health. I expect that the journal will be a vehicle for manuscripts that verify the three conditions of:

(a) assessing an objective hypothesis or pursuing a clear analytical objective;
(b) make use of state-of-the-art analytical methods applied to simulated, field, or experimental data; and
(c) present and discuss results that, ultimately, will lead to actions and recommendations that improve the animal health, welfare, and production, or the safety of animal products globally.

This journal will bring a new breadth of quantitative methods applied to support the health of animal and human populations worldwide. After all, “*probability theory is nothing but common sense reduced to calculus; it makes one appreciate with exactness that which accurate minds feel with a sort of instinct, often without being able to account for it*” (31).

## Conflict of Interest Statement

The author declares that the research was conducted in the absence of any commercial or financial relationships that could be construed as a potential conflict of interest.
